# Expressions of masculinity and associations with suicidal ideation among young males

**DOI:** 10.1186/s12888-020-2475-y

**Published:** 2020-05-12

**Authors:** Tania L. King, Marissa Shields, Victor Sojo, Galina Daraganova, Dianne Currier, Adrienne O’Neil, Kylie King, Allison Milner

**Affiliations:** 1 Centre for Health Equity, Melbourne School of Population and Global Health, The University of Melbourne, Bouverie St, Carlton, 3010 Australia; 2 Centre for Workplace Leadership, Department of Management and Marketing, The University of Melbourne, Melbourne, Victoria 3010 Australia; 3 Longitudinal and Lifecourse Studies, Australian Institute of Family Studies, Honorary Melbourne University Fellow, Southbank, VIC 3006 Australia; 4 Centre for Mental Health, Melbourne School of Population and Global Health, The University of Melbourne, Bouverie St, Carlton, 3010 Australia; 5 Food and Mood Centre, IMPACT Strategic Research Centre, Deakin University, Geelong, 3220 Australia; 6 Turner Institute for Brain and Mental Health, School of Psychological Sciences, Monash University, Clayton, 3800 Australia

**Keywords:** Masculinity, Suicidal ideation, Young males

## Abstract

**Background:**

Adolescent boys and young men are at particular risk of suicide. Suicidal ideation is an important risk factor for suicide, but is poorly understood among adolescent males. Some masculine behaviors have been associated with deleterious effects on health, yet there has been little quantitative examination of associations between masculinity and suicide or suicidal ideation, particularly among boys/young men. This study aimed to examine associations between conformity to masculine norms and suicidal ideation in a sample of adolescents.

**Methods:**

A prospective cohort design, this study drew on a sample of 829 Australian boys/young men from the Australian Longitudinal Study on Male Health. Boys were 15–18 years at baseline, and 17–20 years at follow-up. Masculine norms (Wave 1), were measured using the Conformity to Masculine Norms Inventory (CMNI-22). Suicidal ideation (Wave 2) was a single-item from the Youth Risk Behavior Survey. Logistic regression analysis was conducted, adjusting for available confounders including parental education, Indigenous Australian identity and area disadvantage.

**Results:**

In adjusted models, greater conformity to violent norms (*OR* = 1.23, 95% Confidence Interval [CI]: 1.03–1.47) and self-reliance norms (*OR* = 1.40, 95% CI: 1.15–1.70) was associated with higher odds of reporting suicidal ideation. Greater conformity to norms regarding heterosexuality was associated with reduced odds of reporting suicidal ideation (*OR* = 0.80, 95% CI: 0.68–0.91).

**Conclusions:**

These results suggest that conforming to some masculine norms may be deleterious to the mental health of young males, placing them at greater risk of suicidal ideation. The results highlight the importance of presenting young males with alternative and multiple ways of being a male. Facilitating a relaxation of norms regarding self-reliance, and encouraging help-seeking, is vital. Furthermore, dismantling norms that rigidly enforce masculine norms, particularly in relation to heteronormativity, is likely to benefit the broad population of males, not only those who do not conform to heterosexual and other masculine norms.

## Background

Mental health problems increase during the period of adolescence [47], and for young people aged 10–24 years, mental disorders are the largest contributor to the global burden of disease [25]. Many risk factors and health problems faced by adolescents as they transition through this period are gender specific [3], and on some indicators of mental health, such as suicide, adolescent boys and young men are at particular risk [1]. Despite this, adolescent male health has long been neglected. However there is now growing recognition of the extent to which this population is underserved, particularly in relation to unmet mental health care needs [54].

Globally, suicide is the third most common cause of death for adolescent males [65] and in Australia, suicide is the leading cause of death for males aged 15–24 years [1]. It is often during adolescence that the onset of suicidal and self-harming behaviors occurs [29], and rates of self-injury (a known risk factor for suicide [52];) are typically higher among adolescents than among adults [45]. Research examining risks related to adolescent suicide have typically focused on family factors and psychiatric disorders, but it is recognized that other under-researched factors may also be important and should be investigated [29]. Masculinity, and its associated practices and behaviors is one potential avenue of investigation.

### Conceiving masculinity

Conceptualizations of masculinity have shifted away from the notion of a singular ‘masculinity’, toward recognition of a multiplicity of ‘masculinities’ [14, 32]. Among these multiple masculinities, there exists an idealized or ‘hegemonic’ masculinity that represents the currently accepted ideal [14, 32]. The norms of masculinity are contestable (shifting across time, space and context), as well as relational (hierarchically positioned and performed in relation to femininity and non-hegemonic masculinities [14]). Consistent with this, there is evidence that conformity to masculine norms varies by age [53], and that conformity to masculinity is most strongly endorsed by younger males [53].

The social norms that define appropriate masculine roles and behaviors are assimilated from a young age [7]. The pressure to conform to masculine ideals can be immense [2], and there are often social penalties for boys and men who deviate from normative masculine roles and behaviors [57]. Confinement to the set of behaviors considered to appropriately affirm masculinity can also severely delimit healthy behaviors and emotional responses [2] that might otherwise buffer young males during the often stressful period of adolescence.

### Masculinity among adolescent males

Australia is a diverse country, with many cultural and historical influences shaping its masculine identities. For Australian adolescent males, the physical practice of masculinity is principally located in two key domains: sexuality and sport [15]. For many adolescent Australian boys, sport provides an arena for ‘ritualized combat’ ([15] pg 15), camaraderie and strength. Heterosexuality is central to normative Australian masculinity, and sport has traditionally been a key setting for the display of hetero-masculinity [15]. This is similar for young males elsewhere: a recent systematic review of studies from 29 (mostly Western) countries examining gender attitudes indicated that physical strength, toughness and competitiveness, and heterosexual prowess were central to adolescent masculine norms [34]. Recent evidence indicates that young Australian men are becoming more progressive on some elements of masculinity, with lower endorsement of norms regarding violence, more openness to partaking in traditionally female activities such as household tasks and cooking, and greater openness to having gay friends being recently observed [59]. Some masculine norms however, remain entrenched: many young men retain ideals of ‘acting strong’, being the primary breadwinner and ‘fighting back when pushed around’ [59].

### The gender paradox in suicide

While suicide is a leading cause of death among adolescent males in Australia [1] and worldwide [65], there is little understanding of why. Researchers have also observed evidence of a ‘gender paradox’ in suicide, with males more likely to die by suicide, while females have higher rates of non-fatal suicidal behaviors [9, 38]. Evidence among 14–15 year old Australian adolescents also reflects these international patterns, with more girls than boys reporting suicidal behaviors (including ideation, making a plan and attempting suicide) [20], and more young males dying by suicide [1].

### Ideation-to-action framework

Suicidal ideation is recognized as a putative and proximal risk factor for suicide attempts [37], and contemporary models of suicide recognize this relationship [37, 58, 62]. The process by which suicidal thoughts progress to action is poorly understood, and this lack of understanding is posited to underpin the limited success in reducing suicides worldwide [37]. It is recognized that many commonly cited risk factors for suicide such as depression, hopelessness and impulsivity, also predict suicidal ideation, and do not necessarily differentiate those who have attempted suicide, from those who have reported ideation, but have not attempted [37]. The ideation-to-action framework proposes that the development of ideation, and the progression from ideation to attempts be conceived as distinct processes with separate predictors and explanations [37]. Acquired capability for suicide is considered to be a key factor that may explain the progression from ideation to attempts [33, 37].

There is some evidence that males may be at greater risk of death by suicide because they are socialized to conform to certain masculine norms that foster engagement with painful and provocative life events, resulting in greater ‘acquired capability’ for suicide [26]. It is known that certain physical practices more common among men and boys, such as physical violence and risky behaviors, such as drinking, smoking and dangerous driving, are associated with increased health risks [15, 18]. Given these factors, the effect of masculine norms and socialization on health and health behaviors has become an increasing focus of investigation [12, 13, 49]. It is argued that high conformity to masculine ideals of toughness and emotional neutrality may have harmful effects on the mental health of males [18]. This has prompted calls to apply a masculinities perspective to suicidal behavior [13].

### Masculinity as a risk factor for suicidal ideation

There is an emerging body of work examining masculinity and gender roles as a risk factor for suicidal ideation and suicide in young adults and adolescents [13, 50]. In a large sample of US college students, there was evidence that what the authors termed ‘traditional’ masculinity was associated with suicidal ideation [13]. Psychological autopsies of young men aged 18–30 who had died by suicide in Norway indicated that identifying with unattainable masculine ideals was a key risk factor in these suicides, and death by suicide was theorized to represent an act of masculinity that compensated for this perceived failure to attain idealized masculine standards [50]. Relatedly, among a group of young men aged 18–30 years who had attempted suicide, it was found that conformity to masculine norms about emotional containment prevented young men from disclosing the extent of their distress [12]. Among young men of a similar age (18–24 years), being in a peer group that valued self-reliance and repudiated help-seeking, inhibited help-seeking by young men at risk of suicide, and drove them to adopt risky coping behaviors, such as alcohol use [39]. Consistent findings emerged from a recent meta-analyses, with evidence that certain masculine norms are related to poorer mental health-related outcomes [64].

Not all masculine norms are associated with adverse effects however [64], and it is likely that some dimensions of masculinity are positively associated with mental health and wellbeing. Further, while endorsement of certain masculine norms such as self-reliance is associated with suicidal ideation [49] and poorer mental health in adults [43], less is known about adolescent males.

### Study aims

Suicidal ideation is an understudied phenomena, particularly in relation to masculinity [13] and to our knowledge, no quantitative study has prospectively examined associations between masculinity and suicidal ideation in an Australian population-based sample of adolescent males. The aim of the study was exploratory, and sought to examine associations between conformity to different masculine norms and suicidal ideation among Australian adolescent males. Better understanding of potentially damaging (and health promoting) masculine norms among adolescents is critical if we are to identify ways to promote the mental health and wellbeing of male adolescents and young men.

## Methods

### Study design and setting

We used data from Waves 1 and 2 of the Australian Longitudinal Study on Male Health (*Ten to Men)* [6]. *Ten to Men* is a longitudinal cohort study of Australian boys and men aged 10–55 years at baseline, and collects data on five broad domains (physical health, mental health and wellbeing, health behaviors, social determinants of health, and health service use and knowledge).

Details of the sampling, recruitment, and data collection methods of the *Ten to Men* study have been published elsewhere [19]. The study commenced in 2013/2014 with a cohort of 15,988 males aged 10–55 years. Wave 2 of data collection was conducted between November 2015 and May 2016 with 76% of the original cohort participating. Surveys were self-completed. The current analysis drew on the *Ten to Men* sample of adolescents who were 15–18 years at baseline (Wave 1), and 17–20 years at follow-up.

### Measures

#### Exposure variable: conformity to masculine norms

The Conformity to Masculine Norms Inventory (CMNI-22) was used to assess masculinity and was collected at Wave 1. The CMNI was designed to measure the extent to which males conform to masculine norms. The CMNI-22 is an abbreviated version of the original 94-item scale, using the two highest loading statements to assess conformity to each masculine norm subscale [46]. Pairs of statements correspond to 11 subscales: (1) Primacy of Work; (2) Dominance; (3) Risk-Taking; (4) Heterosexual presentation; (5) Power over Women; (6) Emotional Control; (7) Playboy; (8) Violence; (9) Pursuit of Status; (10) Winning; and (11) Self-Reliance.

It should be noted that in some research, the fourth subscale is referred to as ‘*disdain for homosexuals*’. Following the precedent of other work [49], we refer to this factor as *heterosexual presentation*, noting that the two items used to derive this factor reflect the importance of being perceived to be heterosexual, and a fear of being perceived to be gay, rather than homophobia.

The CMNI instructs respondents to consider their actions, feelings, and beliefs when rating their agreement or disagreement with each statement. Response options range from “strongly disagree” (0) to “strongly agree” (3). Responses to each item were summed to provide a conformity score for each subscale ranging from 0 to 6 [41]. Scores from each of the 11 subscales were summed to present a continuous, global score of conformity to masculine norms from 0 to 66 (higher scores indicating greater conformity to masculine norms). Studies typically report associations using the total CMNI score, however there is evidence that different subscales can be associated with different outcomes, and reliance on the overall score can obfuscate such associations [24]. We therefore examined the different subscales, as well as the overall score. These were analyzed as continuous variables.

#### Outcome variable: suicidal ideation

The primary outcome variable used in this analysis was a single-item from the Youth Risk Behavior Survey, and asked: *Have you seriously thought about killing yourself in the past 12 months?* [10]*.* Responses were coded as a binary variable (yes/no) and collected at Wave 2 only, approximately 2 years after Wave 1 data collection.

#### Covariates

Previous work has shown associations between adolescent suicide/suicidal behaviors and area disadvantage [28], race/ethnicity [38] and Indigenous Australian identity [21]. It is also known that masculinity varies by race/ethnicity [27], and area disadvantage [12]. Given these associations, we included the following covariates as confounders in analytical models: country of birth (Australia, born elsewhere); Indigenous Australian identity (Aboriginal and/or Torres Strait Islander, non-Aboriginal or Torres Strait Islander); area disadvantage. The area disadvantage variable was derived using the Australian Bureau of Statistics’ [4] Index of Relative Socio-Economic Disadvantage (IRSD). IRSD values were categorized into quintiles (based on the sample distribution), with the lowest quintile (Quintile 1) reflecting areas of greatest disadvantage.

### Participants

We restricted the sample to adolescents aged 15–18 years at baseline. As with most cohort studies, there was some loss to follow-up between the two waves: of the 1333 young men aged 15–18 at Wave 1, 960 participated in Wave 2 of data collection. Our eligible sample was defined as those participating in both waves of data collection (*n* = 960). Of these, 935 provided outcome data. There was a small amount of missing data (< 4%) for all exposures, outcomes and covariates, and 7.9% missing for the total CMNI score, reducing our analytic sample to *n* = 829 (see Additional file 1: Table S1).

Comparing the analytic sample with the eligible sample, there was no difference in terms of the masculinity subscales, suicidal ideation, or country of birth. The eligible sample however, contained a slightly higher proportion of Indigenous Australians (4.2% of eligible sample, compared to 3.0% of analytic sample) and a slightly lower proportion were living in an area of least disadvantage (21.8% in eligible sample compared to 24.4% in analytic sample).

**Fig. 1:**
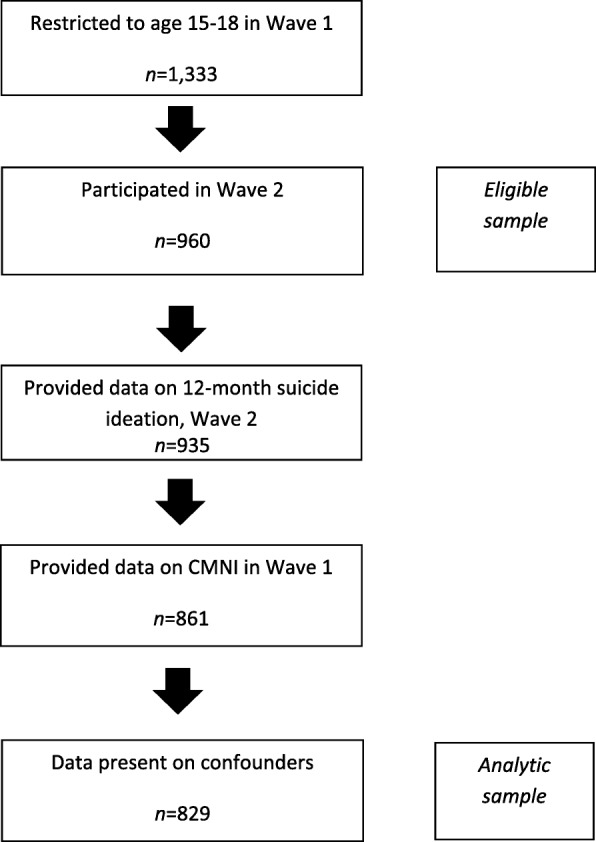
Participants in sample

As for most longitudinal studies, most of the missing data in this analysis was due to participant drop out. While multiple imputation is one of the methods available to address missing data, imputing dependent (outcome) variables does little to improve model efficiency [66]. On this basis, and as most of our missing data was due to missing outcomes, we chose not to use multiple imputation to handle the missing data. Figure 1 illustrates the flow of participants from baseline, through to the eligible and analytic samples.

### Analytical approach

To our knowledge, no previous study has examined the scale properties of the CMNI with Australian adolescents. Given this, we firstly assessed the internal reliability of the CMNI and then conducted confirmatory factor analysis. Descriptive analyses of exposures, confounders, and outcomes were conducted, followed by logistic regression in which models were adjusted for potential confounders (area disadvantage, Indigenous Australian identity, country of birth). As we were interested in the effect of each masculinity subscale, independent of the other subscales, separate regression models were conducted for each masculinity subscale.

### Sensitivity analysis

As it is possible that conformity to masculine norms varies by sexual orientation, we conducted sensitivity analysis in which we adjusted for sexual orientation (heterosexual; gay/ bisexual/other/unsure; see Additional file 1: Table S2 and S3). In further analyses, we also restricted analysis to the sample of young men identifying as heterosexual (See Additional file 1: Table S4).

## Results

We conducted confirmatory factor analysis to assess the factor structure of the CMNI among the adolescent sample. We employed an oblique rotation, as this accommodates the assumed correlated nature of the factors. Following Kaiser criteria, we retained eigenvalues of 1 or higher. In the derived 11-factor structure, item loadings confirmed the factor structure of the CMNI (see Additional file 2: Tables S5 and S6). Internal consistency of the overall CMNI among the adolescent sample was assessed using Guttman’s λ-4, and was calculated to be 0.86.

Table 1 shows the characteristics of the final sample in Wave 1. In this sample, 3.0% were Aboriginal and/or Torres Strait Islander, and for most respondents (66.7%), both parents were born in Australia. Eight percent of the sample reported suicidal ideation within the past 12 months (as measured at Wave 2). The extent to which the sample conformed to masculine norms varied across the different subscales. There was greatest conformity to ‘pursuit of status’, ‘heterosexual presentation’, and ‘emotional control’.

**Table 1:** Sample descriptives (*n* = 829)

	*N*	%
Indigenous Australian Identity		
Aboriginal and/or Torres Strait Islander	25	3.0
Non-Indigenous	804	97.0
Parents’ Country of Birth		
Both born in Australia	554	66.8
One or both born elsewhere	275	33.2
Area disadvantage		
Quintile 1 (Most disadvantaged)	142	17.1
Quintile 2	130	15.7
Quintile 3	184	22.2
Quintile 4	171	20.6
Quintile 5 (Least disadvantaged)	202	24.4
	Mean	Standard Deviation
Conformity to Masculine Norms		
Pursuit of Status	3.50	1.12
Dominance	2.43	1.17
Emotional Control	3.28	1.43
Heterosexual Presentation	3.41	1.74
Playboy	1.65	1.43
Power over Women	1.10	1.05
Primacy of Work	2.99	1.29
Risk Taking	3.02	1.32
Self-Reliance	2.46	1.23
Violence	2.86	1.45
Winning	2.46	1.26
Total Score	29.13	5.87
	*N*	%
12 Month Suicidal Ideation, Wave 2		
Yes	69	8.3
No	760	91.7

Results of the unadjusted and adjusted logistic regression models of the relationship between conformity to masculine norms and suicidal ideation are reported in Table 2. In adjusted models, conformity to violence norms (*OR* = 1.23, 95% CI: 1.03, 1.47; *p* = 0.026), and self-reliance norms (*OR* = 1.40, 95% CI 1.15, 1.70, *p* = 0.001), were both associated with increased odds of reporting suicidal ideation. Greater conformity to heterosexual norms was associated with reduced odds of reporting suicidal ideation (*OR* = 0.80, 95% CI: 0.68, 0.91, *p=* 0.001). There were no associations with suicidal ideation for any of the other masculinity subscales.

**Table 2:** The relationship between conformity to masculine norms (Wave 1) and thoughts of suicide (Wave 2), unadjusted and adjusted logistic regression models (*n* = 829)

	Unadjusted results			Adjusted results^a^		
	*OR*	95% CI	*p* value	*OR*	95% CI	*p* value
Pursuit of Status	1.00	0.80, 1.24	0.974	1.01	0.81, 1.27	0.905
Dominance	1.01	0.81, 1.24	0.961	1.00	0.81, 1.23	0.964
Emotional Control	0.99	0.83, 1.17	0.885	0.98	0.82, 1.17	0.832
Heterosexual Presentation	0.80	0.70, 0.93	0.003	0.80	0.68, 0.91	0.001
Playboy	1.07	0.90, 1.26	0.460	1.07	0.91, 1.27	0.422
Power over Women	0.96	0.75, 1.21	0.706	0.94	0.74, 1.19	0.602
Primacy of Work	0.93	0.76, 1.12	0.435	0.91	0.75, 1.11	0.345
Risk Taking	0.99	0.82, 1.20	0.924	0.99	0.82, 1.19	0.871
Self-Reliance	1.42	1.17, 1.72	< 0.001	1.40	1.15, 1.70	0.001
Violence	1.23	1.03, 1.47	0.025	1.23	1.03, 1.47	0.026
Winning	0.88	0.72, 1.08	0.208	0.88	0.72, 1.07	0.194
CMNI Total	1.00	0.96, 1.04	0.978	1.00	0.96, 1.04	0.868

^a^adjusted for Indigenous Australian identity, parent country of birth, and area disadvantage

While most of the sample identified as heterosexual (*n* = 747), *n* = 63 identified as gay, bisexual, not sure or other. We therefore considered the possibility that associations may vary by sexual orientation (see Additional file 1: Table S2 for sample proportions), and ran models in which we further adjusted for sexual orientation. These produced estimates consistent with the main analyses (see Additional file 1: Table S3). We also restricted analysis to those adolescents identifying as heterosexual and found results consistent with the main analyses (see Additional file 1: Table S4).

## Discussion

This study contributes new understandings of the associations between masculinity and suicidal ideation among adolescent males. Specifically, we found evidence that some dimensions of masculinity were associated with suicidal ideation: notably high conformity to violence and self-reliance among adolescents at 15–18 years was associated with higher odds of reporting suicidal ideation at 17–20 years, and higher conformity to norms related to heterosexuality was associated with lower odds of reporting suicidal ideation. To our knowledge, this is one of the first studies to quantitatively examine the associations between masculinity and suicidal ideation among young males.

The results for violence indicate that each unit increase in conformity to violence was associated with 23% higher odds of reporting suicidal ideation. This is consistent with evidence showing that violent behavior is a risk factor for suicide in adults [17]. While as a whole, the sample did not overwhelmingly endorse violence as a mechanism to solve problems, those who did were more likely to think about suicide, illustrating the risky nature of this dimension of masculinity. There is some evidence that males may be at greater risk of death by suicide because they are socialized to conform to certain masculine norms that foster engagement with painful and provocative life events, resulting in greater ‘acquired capability’ for suicide [26]. It is also known that, compared to females, males are more likely to die by suicide using violent means [61]. Importantly, while hegemonic masculinity values physical strength and toughness, it does not equate with physical violence: violence, however, is sometimes used to demonstrate this physical strength and toughness [8].

The associations observed in this analysis for self-reliance show that each unit increase in self-reliance is associated with 40% increased odds of reporting suicidal ideation. These results are concordant with other work among adults, where self-reliance has been associated with suicidal ideation [49] and mental health problems [43]. The self-reliance items used in this scale tap into affective and behavioral responses to help-seeking. Critically then, these results reveal that some of the young men in this sample reporting suicidal ideation have also reported high conformity to norms that indicate resistance to help-seeking. On face-value, self-reliance can be a positive attribute if it fosters independence, however, the potentially positive effects of self-reliance may be circumscribed if it also inhibits communication and help-seeking in times of distress or crisis. Mental health stigma is known to be a barrier to help-seeking, however recent work highlighted that for young men, this is perhaps more keenly experienced because poor mental health, and help-seeking are both at odds with their internalized masculine norms [39].

Our findings that heterosexual presentation was associated with reduced suicidal ideation (20% lower odds for each unit increase in conformity to heterosexual presentation) was unexpected. The results may evince the protective effect of conforming to socially condoned norms. Hegemonic masculinity is clearly heteronormative. Alignment with dominant masculine norms, and more pointedly, the knowledge that one does not deviate from this heterosexual norm, is likely to confer some level of protection for young males’ mental health. The obverse of the relationship that we observed between high endorsement of heterosexual norms and low suicidal ideation is that low endorsement of heterosexual norms is associated with increased odds of reporting suicidal ideation. Such results do not indicate that being heterosexual is protective, but rather, highlight: firstly, the broader buffering effect of conforming to heterosexual masculine norms; and secondly, the potential to avoid the penalties that arise if deviating from socially accepted norms.

Given that there is a well-established literature documenting the fact that sexual minority young men and adolescents are at elevated risk of suicide and self-harm [20, 51, 55], we conducted sensitivity analysis in which we firstly controlled for sexual orientation, and then restricted our sample to heterosexual-identifying adolescents. The results persisted in both sets of sensitivity analysis. It is widely accepted that heterosexuality is a core component of constructions of hegemonic masculinity [14, 32] and that “*to a greater or lesser extent hegemonic masculinity is constructed as a gender position that is as much ‘not gay’ as it is ‘not female’*” ([32], p. S113). Certainly, the importance of heterosexuality among adolescents in this sample was evidenced by the high mean score for that subscale, although we note that the large standard deviation suggests that this was not uniformly endorsed. Compared to 18–55 year-olds in the same dataset [49], the adolescents in this sample expressed greater conformity to heterosexual norms. The practice of constructing and affirming masculinity through the assertion of heterosexuality among young males has been observed elsewhere [23]. Froyum’s research revealed the ways that adolescent males (and females) disassociate themselves from homosexuality and other non-heterosexual sexual identities to construct and affirm their heterosexuality [23].

Given that they are situated outside the hetero-normative bounds of hegemonic masculinity, it is not surprising that those young males in our sample not conforming to the hegemonic norm of heterosexual presentation fare less well. This is problematic, not only for the negative impact on young people who are not heterosexual, but also because homophobia, and or the fear of being thought to be gay, can act as a barrier to intimacy among men [14]; something that may impart other negative impacts on them in the future, even if not observed now.

These findings suggesting that certain elements of masculinity may place young men at risk of suicidal ideation have implications for suicide prevention programs among adolescent boys. At a broad level, these results indicate the pervasive power of social norms in defining consensual expectations about what group members do, and should do [11]. Previously, gender norms have been examined in relation to the way they delimit the roles, autonomy and control that women have over their lives. Yet, it is increasingly recognized that gender norms and attitudes may also underpin adverse health behaviors and outcomes in boys and men [34, 35]. Recognizing the challenging and often conflicting messages that young adolescents face regarding masculinity is vital [40]. There is clearly a need to destigmatize mental health, and also foster new understandings of masculinity that incorporate help-seeking into masculine ideals [39].

Jewkes et al. [31] proposed an ecological approach to the transformation of masculinities in adolescents. Such an approach seeks to understand and address the drivers of social norms at all levels- societal, institutional (such as schools), interpersonal and individual - and should seek to understand how different factors or identities might intersect [31]. Jewkes et al. [31] proposed that interventions must move away from one dimensional, homogeneous depictions of masculinity. Drawing on Connell’s work [14, 16], they proposed that interventions should emphasize the heterogeneity of masculinity, avoid stereotypes, focus on similarities between men and women, engage with, and acknowledge fears and vulnerabilities and address homophobia [31]. It is possible that relaxing rigid norms regarding masculinity and encouraging acceptance of more diverse masculinities will deliver benefits beyond suicide and mental health improvements for adolescent boys, and contribute to better health and wellbeing in the wider population [22, 36].

Further work is needed to examine the associations observed in this analysis over time (as more waves of data become available), and across different age groups, to understand whether these associations reflect cohort effects, or developmental stages. It is also crucial to understand how these changes relate to other personal, occupational and well-being indicators, regardless of the drivers. Furthermore, it is possible that specific dimensions of masculinity are both protective and also risk factors at different developmental stages: the dynamic nature of these associations needs to be understood.

Dismantling masculine norms to facilitate help-seeking is vital, however it is also important that once adolescent males do seek help, mental health services are available and appropriate to meet their needs – ideally person-centered approaches that acknowledge the diversity of men, and the diversity of their needs [56].

There are several strengths of this analysis. We used a large sample of Australian male adolescents, which strengthens the basis for statistical inference. We also note the use of a validated measure of masculinity. The original 94-item CMNI had good construct validity, and discriminant validity, and the 22-item instrument has been shown to correlate well with the original scale [60]. Additionally, the CMNI-22 has been widely used and is positively regarded. While in this study, basic psychometric parameters of the CMNI were analyzed (i.e. factor structure, item loadings and internal consistency), it should be acknowledged that an exhaustive evaluation of its psychometric properties has not been conducted on an Australian adolescent sample. We also raise the possibility that the construct validity of some of the subscales was low: in particular, it is possible that the items for heterosexual presentation do not align with the construct they are purported to measure in this population (potentially underpinning the findings for heterosexual presentation).

We note that significant measurement differences across ethnic groups have been observed, with evidence that the scale is more theoretically consistent for White American men compared to Asian Americans [30]. Other evidence also indicates that masculinity may be understood, experienced and expressed differently across different ethnic groups and cultural contexts [27], thus the results may not be generalizable to Indigenous Australians and Australians of ethnic minority backgrounds. Further work using qualitative methods is needed to examine how conformity to masculinity may vary across ethnicity and Indigenous identity in Australian adolescents.

As both masculinity and suicidal ideation were self-reported, dependent measurement error, which can arise when two or more variables are based on self-reported subjective responses from the same respondent [63], may have biased findings.

While there is a precedent for the use of single-item suicidal ideation measures [44, 48], there is some evidence that single-item measures may result in a higher proportion of false positives and false negatives [42]. If this occurred in this study, there is potential that it led to some degree of misclassification bias and potentially spurious findings. A further limitation of single-item measures such as this one is that they inadequately capture differences in the severity or frequency of ideation or attempts [42]. It is impossible to ascertain, for example, whether a person was actively planning to engage in suicidal behavior, whether a suicide behavior was stopped, or whether these were simply fleeting and non-serious thoughts.

Selection bias due to missing data potentially affected these results, although we note that for most variables, there was < 4% missing from the eligible sample, and this is unlikely to have biased results. Because our sample comprised of adolescent males (with no parent-reported information on household income or occupation), common socio-economic confounders were either unavailable/not obtained (household income, parental occupation), or not yet realized (educational attainment). We were therefore unable to include these possible confounders of the relationship between masculinity and suicidal ideation in analytical models. This may have introduced some bias, as there is evidence that constructions of masculinity differ across socio-economic position (SEP) [14], and that more disadvantaged groups are at greater risk of suicide [28]. However, we note that while we were unable to control for individual SEP, we did control for area SEP. Given evidence that for adolescent males, enactments of masculinity [12] and suicidal behaviors [5] are known to vary by neighborhood/area deprivation, we contend that by controlling for area SEP we have captured much of the confounding that may have been induced by individual SEP.

## Conclusions

In conclusion, this study presents quantitative evidence of associations between elements of masculinity and suicidal ideation in a sample of adolescents. Among the adolescent males in this sample, we found that high conformity to norms of violence and self-reliance was associated with greater odds of reporting suicidal ideation, while high conformity to norms of heterosexual presentation was associated with reduced odds of reporting suicidal ideation. Maximizing adolescent health is key to optimizing adult health and well-being, and these results highlight the potential importance of presenting multiple ways of being a male among adolescents. Facilitating a relaxation of norms regarding self-reliance to encourage help-seeking is vital, and dismantling heteronormative masculine norms is likely to benefit the broad population of males, not only those who do not conform to heterosexual and other masculine norms.

## Supplementary information

**Additional file 1: Table S1.** Summary of missing data. **Table S2.** Sexual orientation statistics. **Table S3.** The relationship between conformity to masculine norms and thoughts of suicide controlling for sexual orientation, (*n* = 810). **Table S4.** The relationship between conformity to masculine norms and thoughts of suicide, restricted to heterosexual young men, (*n* = 747).

**Additional file 2: Table S5.** Factor loadings based on confirmatory factor analysis of the CMNI among *Ten to Men* sample of Australian males aged 15-18 years. **Table S6.** Concordance between CMNI scales and factor loadings.

## Data Availability

The datasets generated and/or analyzed during the current study are not publicly available due to ethics agreements which preclude this. *Ten to Men* data are available at the Australian Data Archive (https://dx.doi.org/10.26193/V2IVIG.)

## Abbreviations

CI: Confidence interval
CMNI: Conformity to Masculine Norms Inventory
IRSD: Index of Relative Socio-Economic Disadvantage
OR: Odds ratio
SA: Statistical Area
SEP: Socio-economic position
