# Supplementary material for: Expressions of masculinity and associations with suicidal ideation among young males
Source: BMC Psychiatry. 2020 May 12;20:228. doi: 10.1186/s12888-020-2475-y (PMC7218581; doi:10.1186/s12888-020-2475-y)
Supplement: Supplementary file 1 — Additional file 1: Table S1. Summary of missing data. Table S2. Sexual orientation statistics. Table S3. The relationship between conformity to masculine norms and thoughts of suicide controlling for sexual orientation, (n = 810). Table S4. The relationship between conformity to masculine norms and thoughts of suicide, restricted to heterosexual young men, (n = 747). [file 12888_2020_2475_MOESM1_ESM.docx]

**Table S1: Summary of missing data** (as a proportion of eligible sample)

| **Variable** | **% missing from eligible sample (n=960)** |
| --- | --- |
| ***Confounding variables (measured at Wave 1)*** | |
| Indigenous Australian Identity | 1.3 |
| Parent country of birth | 3.0 |
| Area disadvantage | 0.0 |
| ***Exposure variables*** |  |
| Pursuit of Status | 0.0 |
| Dominance | 2.0 |
| Emotional Control | 1.6 |
| Heterosexual Presentation | 3.3 |
| Playboy | 3.1 |
| Power over Women | 2.1 |
| Primacy of Work | 1.8 |
| Risk Taking | 2.0 |
| Self-Reliance | 1.9 |
| Violence | 2.6 |
| Winning | 2.1 |
| CMNI | 7.9 |
| ***Outcome variables (measured at Wave 2)*** |  |
| Suicidal Ideation | 2.6 |

Supplementary Table S2: Sexual orientation statistics (n=810)

|  | **N** | **%** |
| --- | --- | --- |
| **Sexual Orientation** |  |  |
| Heterosexual | 747 | 92.22 |
| Gay/Bisexual/Other/Unsure | 63 | 7.78 |
| Total | 810 | 100.00 |

Supplementary Table S3. The relationship between conformity to masculine norms and thoughts of suicide, controlling for sexual orientation: unadjusted and adjusted logistic regression models (n=810)

|  | **Unadjusted results** | | | **Adjusted results*** | | |
| --- | --- | --- | --- | --- | --- | --- |
|  | **OR** | **95% CI** | **p value** | **OR** | **95% CI** | **p value** |
| Pursuit of Status | 0.99 | 0.79, 1.23 | 0.902 | 1.01 | 0.81, 1.26 | 0.916 |
| Dominance | 1.00 | 0.81, 1.23 | 0.994 | 0.97 | 0.78, 1.21 | 0.771 |
| Emotional Control | 0.99 | 0.83, 1.17 | 0.884 | 1.03 | 0.86, 1.22 | 0.780 |
| Heterosexual Presentation | 0.80 | 0.70, 0.93 | 0.003 | 0.82 | 0.71, 0.95 | 0.008 |
| Playboy | 1.07 | 0.90, 1.26 | 0.458 | 1.07 | 0.90, 1.27 | 0.463 |
| Power over Women | 0.96 | 0.75, 1.22 | 0.721 | 0.95 | 0.74, 1.21 | 0.654 |
| Primacy of Work | 0.93 | 0.77, 1.13 | 0.461 | 0.90 | 0.74, 1.09 | 0.285 |
| Risk Taking | 0.99 | 0.82, 1.19 | 0.897 | 1.01 | 0.84, 1.22 | 0.931 |
| Self Reliance | 1.42 | 1.17, 1.72 | <0.001 | 1.40 | 1.15, 1.70 | 0.001 |
| Violence | 1.23 | 1.03, 1.47 | 0.024 | 1.26 | 1.05, 1.51 | 0.015 |
| Winning | 0.88 | 0.72, 1.07 | 0.193 | 0.91 | 0.74, 1.11 | 0.336 |
| CMNI Total | 1.00 | 0.96, 1.04 | 0.995 | 1.01 | 0.96, 1.05 | 0.818 |

*Adjusted for Indigenous Australian identity, parent country of birth, area disadvantage, sexual orientation

Supplementary Table S4. The relationship between conformity to masculine norms and thoughts of suicide restricted to **heterosexual young men**: unadjusted and adjusted logistic regression models (n=747)

|  | **Unadjusted results** | | | **Adjusted results*** | | |
| --- | --- | --- | --- | --- | --- | --- |
|  | **OR** | **95% CI** | **p value** | **OR** | **95% CI** | **p value** |
| Pursuit of Status | 1.06 | 0.82, 1.35 | 0.670 | 1.08 | 0.84, 1.38 | 0.565 |
| Dominance | 1.08 | 0.86, 1.36 | 0.528 | 1.07 | 0.85, 1.35 | 0.575 |
| Emotional Control | 1.01 | 0.83, 1.22 | 0.938 | 1.00 | 0.82, 1.21 | 0.975 |
| Heterosexual Presentation | 0.87 | 0.74, 1.01 | 0.073 | 0.84 | 0.72, 0.99 | 0.032 |
| Playboy | 1.11 | 0.93, 1.34 | 0.244 | 1.13 | 0.94, 1.36 | 0.193 |
| Power over Women | 1.00 | 0.77, 1.30 | 0.992 | 0.97 | 0.75, 1.26 | 0.831 |
| Primacy of Work | 0.92 | 0.74, 1.14 | 0.430 | 0.90 | 0.73, 1.13 | 0.368 |
| Risk Taking | 1.02 | 0.83, 1.26 | 0.864 | 1.00 | 0.81, 1.23 | 0.988 |
| Self Reliance | 1.35 | 1.09, 1.66 | 0.006 | 1.30 | 1.05, 1.61 | 0.017 |
| Violence | 1.21 | 1.00, 1.47 | 0.054 | 1.21 | 1.00, 1.47 | 0.055 |
| Winning | 0.88 | 0.70, 1.10 | 0.250 | 0.88 | 0.70, 1.10 | 0.250 |
| CMNI Total | 1.02 | 0.97, 1.06 | 0.506 | 1.01 | 0.96, 1.06 | 0.691 |

*Adjusted for Indigenous Australian identity, parent country of birth, area disadvantage
