# Supplementary material for: Expressions of masculinity and associations with suicidal ideation among young males
Source: BMC Psychiatry. 2020 May 12;20:228. doi: 10.1186/s12888-020-2475-y (PMC7218581; doi:10.1186/s12888-020-2475-y)
Supplement: Supplementary file 2 — Additional file 2: Table S5. Factor loadings based on confirmatory factor analysis of the CMNI among Ten to Men sample of Australian males aged 15-18 years. Table S6. Concordance between CMNI scales and factor loadings. [file 12888_2020_2475_MOESM2_ESM.docx]

| **Supplementary Table S5:** Factor loadings based on confirmatory analysis of the CMNI among the *Ten to Men* sample of Australian males aged 15-18 years | | | | | | | | | | | | |
| --- | --- | --- | --- | --- | --- | --- | --- | --- | --- | --- | --- | --- |
| **Variable** | **Factor1** | **Factor2** | **Factor3** | **Factor4** | **Factor5** | **Factor6** | **Factor7** | **Factor8** | **Factor9** | **Factor10** | **Factor11** | **Uniqueness** |
| CMNI01 | -0.02 | 0.26 | 0.04 | 0.02 | -0.04 | 0.01 | -0.01 | -0.05 | -0.12 | **0.38** | -0.14 | 0.70 |
| CMNI02 | -0.01 | **0.59** | -0.02 | -0.01 | -0.04 | -0.03 | 0.01 | 0.06 | 0.03 | 0.12 | 0.01 | 0.66 |
| CMNI03 | 0.00 | -0.10 | 0.00 | 0.02 | 0.06 | -0.01 | **0.70** | 0.03 | 0.00 | 0.07 | 0.02 | 0.49 |
| CMNI04 | -0.10 | 0.01 | 0.02 | -0.02 | 0.07 | **0.70** | -0.01 | 0.00 | 0.02 | 0.02 | 0.02 | 0.50 |
| CMNI05 | 0.06 | 0.17 | -0.02 | 0.01 | **0.49** | 0.11 | 0.02 | -0.02 | -0.01 | -0.04 | -0.10 | 0.55 |
| CMNI06 | -0.01 | 0.02 | **0.83** | -0.02 | 0.01 | 0.01 | -0.01 | -0.02 | -0.03 | -0.04 | 0.02 | 0.33 |
| CMNI07 | **0.75** | -0.01 | -0.03 | 0.01 | -0.05 | 0.07 | 0.00 | 0.02 | 0.01 | 0.01 | 0.03 | 0.44 |
| CMNI08 | 0.10 | -0.05 | -0.01 | 0.01 | -0.05 | **0.71** | 0.01 | 0.01 | -0.02 | 0.00 | -0.01 | 0.50 |
| CMNI09 | -0.02 | -0.04 | 0.01 | -0.01 | 0.02 | -0.02 | -0.02 | **0.56** | 0.06 | 0.05 | 0.09 | 0.66 |
| CMNI10 | 0.01 | -0.04 | **0.79** | 0.02 | -0.01 | 0.00 | 0.01 | 0.03 | 0.05 | 0.04 | 0.00 | 0.31 |
| CMNI11 | 0.05 | **0.54** | -0.03 | 0.01 | 0.12 | 0.03 | -0.03 | -0.03 | 0.10 | -0.01 | 0.22 | 0.59 |
| CMNI12 | 0.01 | 0.11 | 0.02 | 0.11 | -0.08 | 0.01 | 0.06 | -0.03 | -0.12 | -0.09 | **0.44** | 0.73 |
| CMNI13 | 0.03 | 0.10 | 0.00 | 0.00 | -0.02 | 0.02 | 0.04 | **0.54** | -0.07 | -0.10 | -0.09 | 0.63 |
| CMNI14 | 0.02 | 0.02 | -0.02 | 0.02 | 0.01 | 0.01 | 0.01 | -0.03 | 0.02 | **0.53** | 0.02 | 0.72 |
| CMNI15 | -0.01 | -0.09 | -0.04 | **0.57** | -0.01 | 0.01 | -0.01 | -0.01 | 0.05 | 0.07 | 0.09 | 0.67 |
| CMNI16 | **0.73** | 0.01 | 0.03 | -0.01 | 0.08 | -0.08 | -0.01 | -0.02 | 0.00 | 0.00 | -0.02 | 0.43 |
| CMNI17 | -0.02 | 0.12 | 0.02 | 0.11 | 0.00 | 0.00 | -0.01 | 0.03 | -0.10 | 0.03 | **0.41** | 0.76 |
| CMNI18 | 0.04 | 0.05 | 0.05 | -0.03 | -0.04 | -0.01 | 0.06 | -0.01 | **0.57** | 0.02 | -0.08 | 0.64 |
| CMNI19 | 0.00 | 0.08 | 0.00 | -0.02 | -0.05 | 0.01 | **0.70** | -0.02 | 0.01 | -0.07 | 0.01 | 0.48 |
| CMNI20 | 0.05 | -0.07 | 0.02 | 0.00 | **0.56** | -0.03 | 0.00 | 0.03 | -0.04 | 0.01 | 0.02 | 0.68 |
| CMNI21 | 0.03 | 0.08 | 0.04 | **0.56** | 0.04 | 0.00 | 0.01 | 0.01 | -0.01 | -0.05 | -0.04 | 0.60 |
| CMNI22 | -0.04 | 0.08 | -0.01 | 0.07 | -0.01 | 0.01 | -0.05 | 0.01 | **0.52** | -0.07 | -0.05 | 0.65 |

According to the terms of use of the scale, we are unable to document the item names. The following table shows which items load onto each factor, and highlight the alignment between the official scale items, and the items loading onto each factor/scale in this dataset.

| **Supplementary Table S6: Concordance between CMNI scales and factor loadings** | | | |
| --- | --- | --- | --- |
| ***Factor Number*** | ***Scale name*** | ***CMNI scale items*** | ***Current sample item loadings*** |
| Factor 1 | Playboy | 7 & 16 | 7 & 16 |
| Factor 2 | Dominance | 2 & 11 | 2 & 11 |
| Factor 3 | Emotional Control | 6 & 10 | 6 & 10 |
| Factor 4 | Winning | 15 & 21 | 15 & 21 |
| Factor 5 | Power over women | 5 & 20 | 5 & 20 |
| Factor 6 | Heterosexual presentation | 4 & 8 | 4 & 8 |
| Factor 7: | Risk-taking | 3 & 19 | 3 & 19 |
| Factor 8 | Violence | 9 & 13 | 9 & 13 |
| Factor 9 | Self-reliance | 18 & 22 | 18 & 22 |
| Factor 10 | Primacy of work | 1 & 14 | 1 & 14 |
| Factor 11 | Pursuit of status | 12 & 17 | 12 & 17 |
